# A Fast and Practical Yeast Transformation Method Mediated by *Escherichia coli* Based on a Trans-Kingdom Conjugal Transfer System: Just Mix Two Cultures and Wait One Hour

**DOI:** 10.1371/journal.pone.0148989

**Published:** 2016-02-05

**Authors:** Kazuki Moriguchi, Shinji Yamamoto, Yuta Ohmine, Katsunori Suzuki

**Affiliations:** Department of Biological Science, Graduate School of Science, Hiroshima University, 1-3-1 Kagamiyama, Higashi-Hiroshima 739–8526, Japan; CNR, ITALY

## Abstract

Trans-kingdom conjugation is a phenomenon by which DNA is transferred into a eukaryotic cell by a bacterial conjugal transfer system. Improvement in this method to facilitate the rapid co-cultivation of donor bacterial and recipient eukaryotic cell cultures could make it the simplest transformation method, requiring neither isolation of vector DNA nor preparation of competent recipient cells. To evaluate this potential advantage of trans-kingdom conjugation, we examined this simple transformation method using vector combinations, helper plasmids, and recipient *Saccharomyces cerevisiae* strains. Mixing donor *Escherichia coli* and recipient *S*. *cerevisiae* overnight cultures (50 μL each) consistently yielded on the order of 10^1^ transformants using the popular experimental strain BY4742 derived from S288c and a shuttle vector for trans-kingdom conjugation. Transformation efficiency increased to the order of 10^2^ using a high receptivity trans-kingdom conjugation strain. In addition, either increasing the amount of donor cells or pretreating the recipient cells with thiols such as dithiothreitol improved the transformation efficiency by one order of magnitude. This simple trans-kingdom conjugation-mediated transformation method could be used as a practical yeast transformation method upon enrichment of available vectors and donor *E*. *coli* strains.

## Introduction

Trans-kingdom conjugation (TKC) enables transfer of a vector DNA cloned and amplified in *Escherichia coli* into *Saccharomyces cerevisiae* directly using a bacterial conjugal transfer system based on the Type-4 Secretion System (T4SS). In a donor cell, the transfer starts from a site called the origin of transfer (*oriT*) located on a TKC vector. Mob proteins nick and disentangle one strand of the vector DNA. Genes for Mob proteins are encoded in either the vector or a helper plasmid. The disentangled liner ssDNA is transferred into a recipient cell via T4SS, which establishes physical contact with the recipient cell and secretes the DNA from the donor. Genes for T4SS are encoded in the helper plasmid [1, 2]. While the influence of TKC on eukaryotic evolution is still controversial [1], this system is attractive for potential applications to yeast transformation methodology. A recent study of this system showed that the transfer of vector plasmids occurs accurately and allows for the excision of unnecessary DNA vector regions during the transfer [2]. While these characteristics are necessary conditions for developing TKC as a gene introduction method, the system is still insufficient for adoption as a preferred method among researchers.

In previous studies, the TKC reaction was performed on solid, non-selective medium for 5–12 h [3, 4]. Bates *et al*. later found that a 1-h reaction time is sufficient when combining a vector bearing the IncP1α-type *oriT* and a helper plasmid with the IncP1α-type conjugal transfer system [5]. Recent studies have further shown that the IncQ-type plasmid vector also transfers quickly, and the reaction is applicable under liquid condition with middle pH buffers [1, 6]. These findings exhibit two potential advantages of TKC-mediated gene introduction: time-saving and simplicity.

When performing transformation reactions with multiple samples, if transformants are consistently obtained, a smaller reaction scale and fewer manipulation steps are generally preferred over high transformation efficiency. Using TKC, a recent study reported that 1 h was sufficient to obtain transformants consistently from a mixture of a minute lump of recipient yeast BY4742 cells and a donor cell suspension (25 μL) containing 3.8 × 10^6^ cfu. However, the experiment used cells in the growing stage, requiring adjustment of the cell concentration, and included a step involving transfer of a donor suspension from its growth medium to a middle pH buffer [1]. A simplified application of the TKC method has not yet been reported.

This study aims to develop a simplified method for the use of TKC. We mixed a small volume of *E*. *coli* stationary phase liquid overnight cultures directly with recipient yeast and evaluated transformation efficiency. We propose an improved TKC-mediated transformation method that shows higher transformation efficiency.

## Materials and Methods

### *E*. *coli* and *S*. *cerevisiae* strains and plasmids

Donor *E*. *coli* strains, recipient *S*. *cerevisiae* strains, and the plasmids used in this study are detailed in Table 1. The primary donor strain used was HB101 bearing the vector pAY205, which carries the IncQ-type plasmid RSF1010 backbone, and the helper plasmid pRH210, which carries an IncP1α-type plasmid RK2 (RP4) conjugal transfer system. YNN281α is isogenic with YNN281 except at the mating type locus. The strain is a MATα segregant arising from the diploid strain YNN281a/α, in which diploidy was induced by the homotalism gene HO as described by Suzuki and Yanagishima [9].

**Table 1:** Bacterial strains and plasmids used in this study.

Strains and plasmids	Relevant characteristics	Source or reference
**Strains**		
***S*. *cerevisiae***		
BY4742	S288c derivative; *MAT*α *his3* Δ*1 leu2*Δ*0 lys2*Δ*0 ura3*Δ*0*	Invitrogen
YNN281α	Mating type transformant of YNN281, same genotype as YNN282; *MAT*α *ade2-1 ura3-52 lys2-801 his3*-Δ*200 trp1-*Δ*1 ssd1-d*	This study
NCYC3623	Fermentation yeast L-1374 derivative; *MAT*α *ho*::*hygMX ura3*::*kanMX-Barcode*	NCYC
NCYC3625	Fermentation yeast DBVPG 6044 derivative; *MAT*α *ho*::*hygMX ura3*::*kanMX-Barcode*	NCYC
NCYC3627	Wild yeast UWOPS03-461.4 derivative; *MAT*α *ho*::*hygMX ura3*::*kanMX-Barcode*	NCYC
NCYC3630	Fermentation yeast Y12 derivative; *MAT*α *ho*::*hygMX ura3*::*kanMX-Barcode*	NCYC
NCYC3631	Wild yeast YPS606 derivative; *MAT*α *ho*::*hygMX ura3*::*kanMX-Barcode*	NCYC
***E*. *coli***		
HB101	*F*^-^ *hsdS20(r*^-^_*B*_ *m*^-^_*B*_*) recA13 ara-14 proA2 lacY1 galK2 rpsL20 xyl-5 mtl-1 supE44 λ*^-^ *leu thi*	NBRP Japan
DH10B	*F*^-^ *mcr*A Δ(*mrr*-*hsd*RMS-*mcr*BC) Φ80*lac*ZΔM15 Δ*lac*X74 *rec*A1 *end*A1 *ara*D139 *Δ*(*ara leu*) 7697 *gal*U *gal*K *rps*L *nup*G *λ*^-^	Invitrogen
S17-1 λ*pir*	*F*^-^ *hsdR recA pro thi* [RP4-2 Tc::Mu Km::Tn7 (Tp Sm)] λ*pir*	NBRP Japan
**Plasmids**		
**TKC vectors**		
pAY205	*oriV*^Q^ *oriT*^Q^ *mob*^Q^ *URA3 TRP1 ARS1* Kan^R^ Tet^R^	*AB526841 [7]
pRS316::*oriT*^P^	Mobilizable plasmid; *URA3 CEN6/ARSH4 ori*-pMB1 Amp^R^ *oriT*^P1α^	[1]
**Helper plasmids**		
pRH210	*tra*^P1α^ *trb*^P1α^ *or*i-pMB1 Amp^R^	*AB526839 [4]
pRH220	*tra*^P1α^ *trb*^P1α^ *ori*-pSC101 Cm^R^	*AB526840 [8]
pDPT51	Helper plasmid; *tra*^P1β^ *trb*^P1β^ *ori*-ColE1 Tp^R^ Amp^R^	Y. Fujita

*DDBJ/EMBL/GenBank accession number

### *E*. *coli* and *S*. *cerevisiae* culture conditions

Donor *E*. *coli* strains were cultured in 3 mL of liquid Luria Bertani (LB) broth with appropriate antibiotics overnight (12–15 h) at 37°C. LB+1.5% agar was used alternatively when the donor was inoculated on solid medium. Recipient *S*. *cerevisiae* strains were cultured in 3 mL of liquid yeast-extract/peptone/dextrose (YPD) broth for one day (18–22 h) at 28°C. Yeast extract-peptone/adenine/dextrose (YPAD) broth was used alternatively if a recipient strain was auxotrophic for adenine. For cultures used in the adjusted trans-kingdom conjugation reaction, donor *E*. *coli* overnight culture (100–150 μL) was inoculated into 3 mL of fresh medium and cultured for 3–4 h to recover cell growth. In parallel, recipient yeast cells were streaked onto fresh YPD+2% agar medium and cultured for 1 day.

### Simple trans-kingdom conjugation

**Fig 1:**
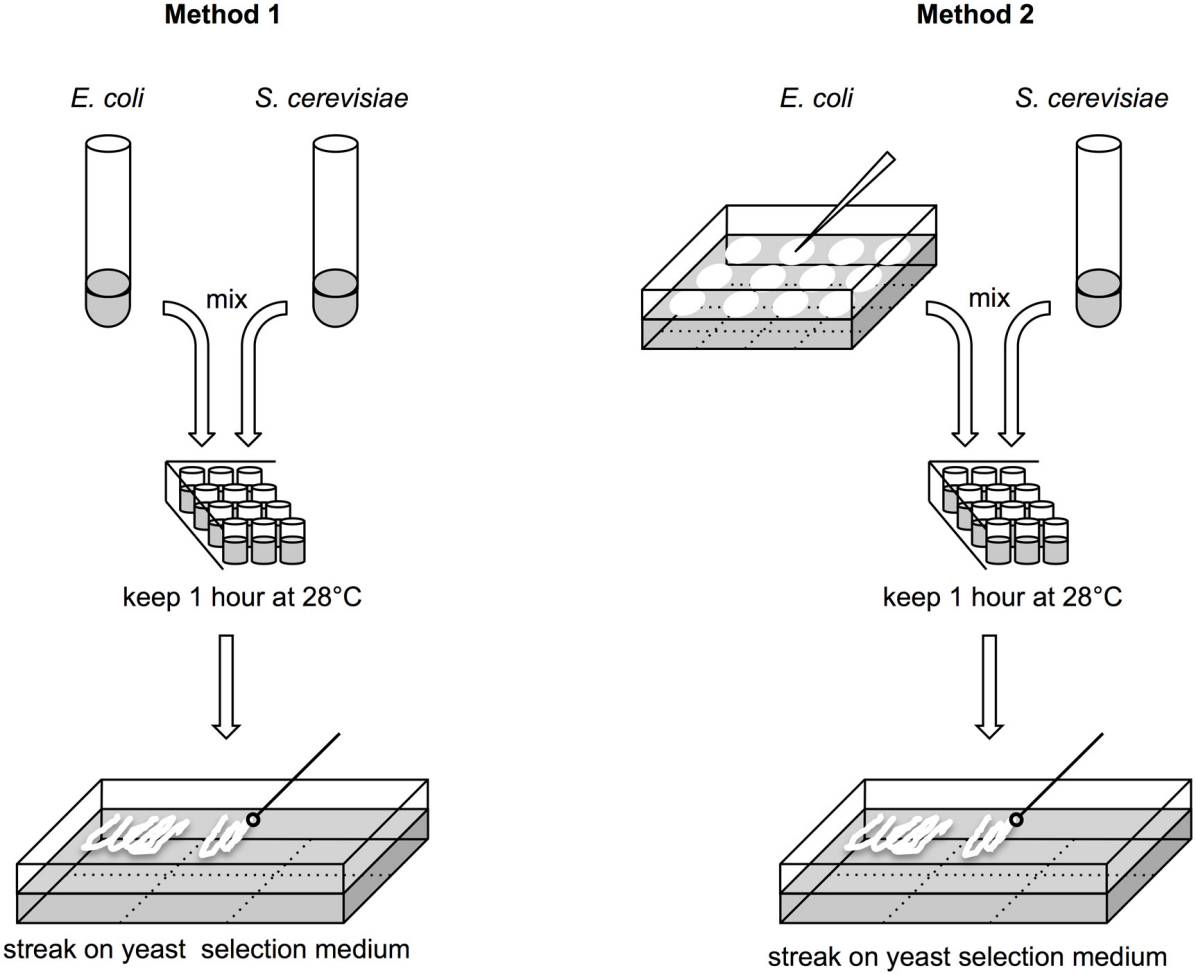
Schematic flow of two simple methods for yeast transformation. For method 1, donor *E*. *coli* bearing a vector and recipient *S*. *cerevisiae* stationary phase cultures (50 μL each) were mixed. In method 2, the donor *E*. *coli* was changed to a solid culture. The donor was picked up with a toothpick and mixed with 50 μL of recipient *S*. *cerevisiae* stationary phase culture.

Schematic flow of the method is shown on Fig 1. Each 50 μL of donor *E*. *coli* and recipient yeast liquid saturated cultures were mixed and incubated for 1 h at 28°C.

In case of using concentrated donor cells, 500 μL of the donor culture was centrifuged, and then 450 μL of supernatant was removed. The remaining culture of 50 μL was resuspended and was served for TKC reaction. The reaction mix was then gently resuspended, and 25 μL of it was then streaked on a plate with synthetic complete (SC) medium without uracil and incubated at 28°C for three to five days. In parallel, to calculate the recipient cell number included in the reaction, 10 μL of 1000-fold to 4000-fold diluted reaction mix was streaked on a YPD (or YPAD) plate. Appropriate antibiotics were added in each plate.

When donor cells grown on solid LB medium were directly used for TKC, cells were picked by toothpick and suspended in 50 μL of recipient culture. After the TKC reaction, 50 μL of TNB was added to the reaction mixture, and 25 μL of the mixed suspension were streaked on a solid selection plate.

As for dithiothreitol (DTT) treatment, 1 M DTT solution was added into 50 μL of yeast recipient culture at a final concentration of 100 mM and incubated for 30 min at 28°C. After collecting cells by centrifugation, cells were re-suspended in 50 μL of YPD and served for the TKC reaction.

### Adjusted trans-kingdom conjugation

The reaction was performed as previously described [1]. Briefly, cells in the growing stage collected from liquid LB and solid YPD (or YPAD if necessary) cultures were used for the donor and recipient, respectively.

Furthermore, 25 μL of TNB buffer (80 mM Tris-HCl [pH 7.5] and 0.05% NaCl) containing 3.8 × 10^6^ cfu of donor and 1.0×10^6^ cfu of recipient were incubated at 28°C for 1 h and plated on selection medium. In case of pretreatment by chemical reagents, recipient cells collected from solid medium were incubated in 50 μL of TNB including each thiol for 1 h at 28°C. The mixture was re-suspended three times during the incubation. After the treatment, cells were collected and suspended with fresh TNB without thiol, and the concentration was adjusted to 1.0 × 10^6^ cfu/12.5 μL by measuring turbidity. Then, 12.5 μL of the thiol treated recipient suspension was mixed with the same volume of the donor suspension containing 3.8 × 10^6^ cfu and TKC reaction was performed. Thiols used in this study were DTT, 2-aminoethanethiol (AET), and 3-mercapto-1, 2-propanediol (MPD) at 100 mM. All chemicals were obtained from Wako Pure Chemical Ind., Ltd. (Osaka, Japan).

### Statistical analysis

Statistical comparison of the transformation efficiency of vector and helper plasmid combinations or strains was performed based on the log_10_-converted value of each TKC efficiency. This method was used due to the wide range of TKC efficiency observed in trials (S1 Fig). All statistical analyses were performed using either Microsoft Excel or the public domain R program (http://www.r-project.org/). The statistical tests used for data analysis and the number of experimental replicates are shown in figure legends and S1 Table.

## Results

### Applicability of simple TKC method in various helper, vector, donor, and recipient combinations

**Fig 2:**
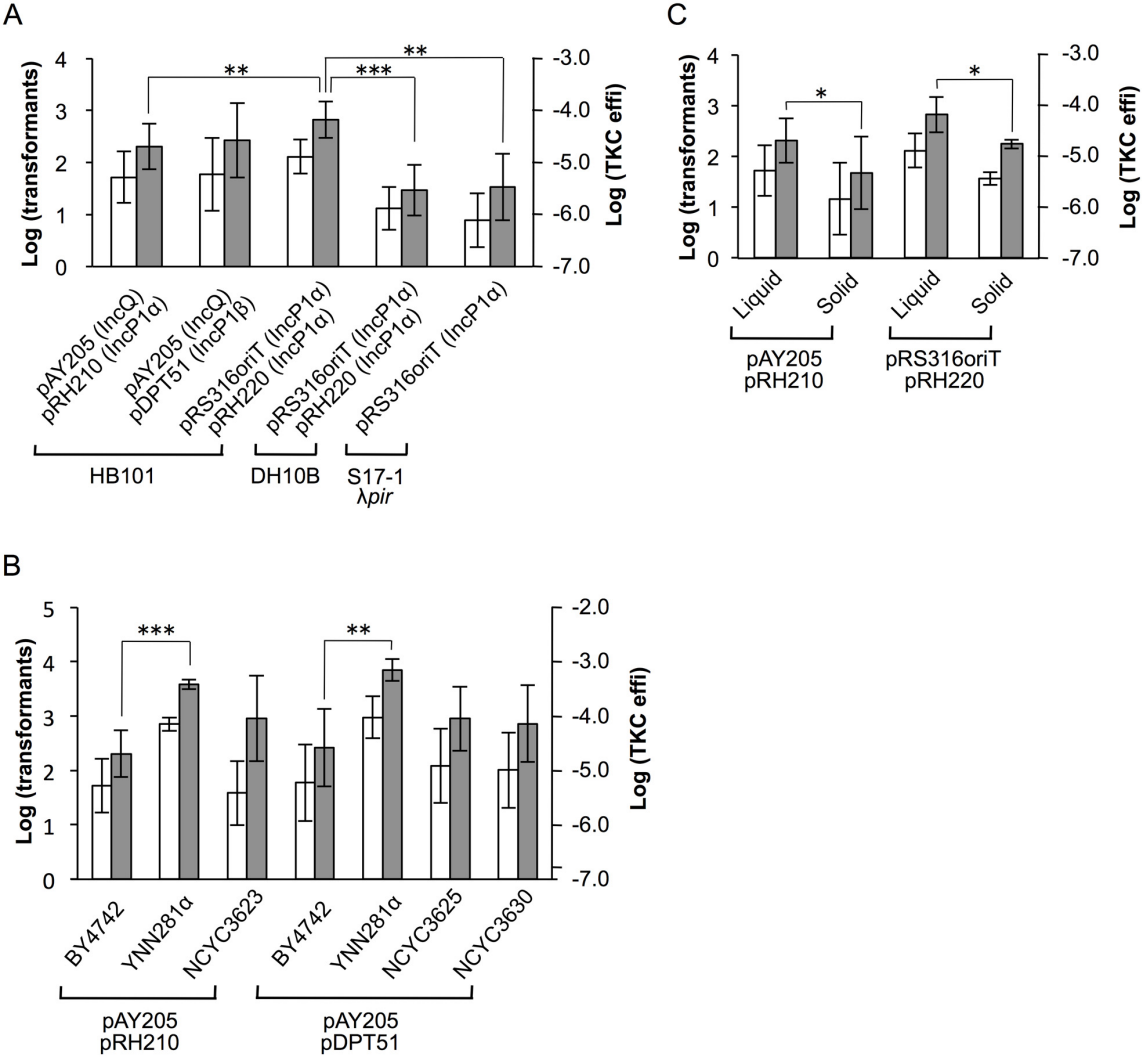
Evaluation of the simple TKC-mediated transformation method. **(A)** Evaluation of the transformation method with various donors. pAY205 and pRS316oriT (represents pRS316::*oriT*^P^) are TKC vectors. pRH210, pDPT51, and pRH220 are helper plasmids. Host *E*. *coli* strains are shown at the bottom. **(B)** Evaluation of the transformation method with various recipients. Recipient *S*. *cerevisiae* strains are shown under the value bars, and the vector and helper combination is shown at the bottom. HB101 served as the host. The far left lane, shown as “BY4742,” is from the same data set used in Fig 2A. **(C)** Evaluation of the transformation method between liquid- and solid-cultured donors. Lanes labeled “liquid” represent results obtained by “Method 1” in Fig 1; data set is the same as that used in Fig 2A. Lanes labeled as “solid” represent results obtained by “Method 2” in Fig 1. The vector and helper combination is shown at the bottom. HB101 served as the host. White bars represent Log_10_ converted value of transformants/reaction, and the scale is shown on the left side of each graph as “Log (transformants)”. Gray bars represent the Log_10_ converted value of transformation efficiency (transformants/recipient cells), and the scale is shown on the right side of each graph as “Log (TKC effi)”. Data are represented as the mean ± SD. Number of trials of each experiment are shown in S1 Table. Asterisks indicate statistically significant differences in two-tailed *t*-test: *p < 0.05, **p < 0.01, and ***p < 0.001.

Using the vector/helper plasmid combination of pAY205 and pRH210, transformants were consistently obtained from BY4742, a derivative of the widely used experimental strain S288c (Fig 2A). Transformants were produced on the order of 10^1^ per reaction. The range of transformants per reaction was 4–272 over 16 experiments using this vector and helper combination (S1 Table).

This method was also successful using pDPT51, which carries the IncP1β-type plasmid R751 conjugal transfer system, in place of the helper plasmid and using the vector pRS316::*oriT*^P^ carrying the IncP1α-type origin of transfer (*oriT*^P^) and another IncP1α helper plasmid pRH220 derived from the RK2 plasmid, demonstrating the broad applicability of this method (Fig 2A). While there was no statistically significant difference between the IncP1α (pRH210) and IncP1β (pDPT51) helper plasmids, the pRS316::*oriT*^P^ (IncP1α) vector demonstrated a higher transformation efficiency than the pAY205 (IncQ) vector. This tendency was also observed using another recipient strain, YNN281α (S1 Table).

To know the preference of donor strain, two *E*. *coli* strains were examined in addition to HB101. DH10B is a commercial strain and is widely used as well as HB101. S17-1 λ*pir* has been used as a donor strain for bacterial conjugation experiments [10] and is available from several bioresource banks. In this strain, conjugal transfer system genes of the RK2 plasmid are integrated into the host chromosome. Thus, if this strain shows constant TKC activity, use of a helper plasmid is not required.

As shown in Fig 2A and S1 Table, pRS316::*oriT*^P^ was successfully introduced into BY4742 from these two strains, although the transformation efficiency was lower than that of HB101 by one order of magnitude. pAY205 was also successfully introduced from the S17-1 λ*pir* donor, but the transfer was below the detection threshold in a trial (S1 Table).

Of several yeast strains transformed using this simple TKC method, YNN281α possessing a defective allele (*ssd1-d*) in TKC blocking [1] had the highest transformation efficiency, regardless of the vector/helper combination (Fig 2B, S1 Table). This strain consistently yielded on the order of 10^2^ transformants per reaction (100 μL), and the maximum number of transformants per reaction was 3692 using pAY205 and pDPT51. This simple method was also applicable to other strains of *S*. *cerevisiae*, which were derivatives of wild and industrial strains. NCYC3623 (Fig 2B), NCYC3625, and NCYC3630 yielded TKC transformants on the order of 10^1^ to 10^2^. NCYC3627 and NCYC3631 also yielded transformants, although they did not yield consistently at this 100 μL reaction scale (S1 Table).

### Modification of the TKC method for further simplification or higher transformation efficiency

We reasoned that if donor *E*. *coli* samples grown on solid medium from frozen stocks could be used directly in recipient yeast suspensions for the TKC reaction, this method would be more practical for multi-sample use. To test the feasibility of this method, donor *E*. *coli* samples were picked up from solid medium with a toothpick and mixed with 50 μL of recipient suspension (in Fig 1, Method 2). The donor successfully transformed the recipients on the order of 10^1^ (Fig 2C). pRS316::*oriT*^P^ yielded a higher transformation efficiency than pAY205, as was observed in liquid donors, although this difference was not statistically significant. The donor cells from solid medium showed lower transformation efficiency by one order of magnitude.

We next tried to increase the transformation efficiency of this system by modifying the preparation method used for either donor or recipient cells.

**Fig 3:**
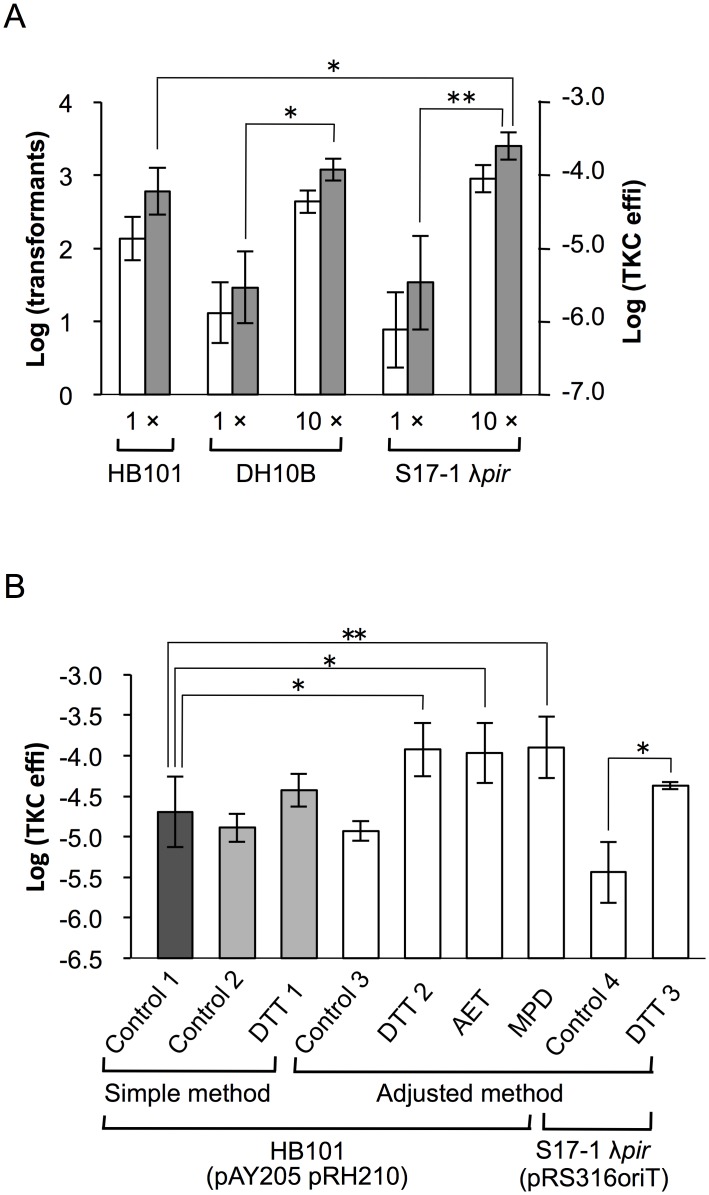
Modification of the TKC method to increase transformation efficiency. **(A)** Effect of increasing the number of donor *E*.*coli* on TKC-mediated transformation. Lanes labeled “1×” represent results obtained using standard number of donor cells (same data sets as Fig 2A); and lanes labeled “10×” represent results obtained using 10 times the number of donor cells. Host strains are indicated at the bottom. pRS316::*oriT*^P^ was used as the TKC vector, and pRH220 was used as the helper plasmid in DH10B. White bars, log_10_ converted value of transformants/reaction; Gray bars, log_10_ converted value of transformation efficiency (transformants/recipient cells). Data are presented as mean ± SD. Number of trials of each experiment is shown in S1 Table. **(B)** Effect of thiol treatment of recipient yeast on TKC-mediated transformation. Lanes labeled as “DTT,” “AET” and “MPD” indicate recipient yeast samples pretreated with dithiothreitol, 2-Aminoethanethiol, and 3-Mercapto-1,2-propanediol, respectively. Lanes labeled as “Control” indicate non-treated controls. Control 1 is the same data set used in Fig 2A (dark gray bar). Control 2 (light gray bar), Control 3 and Control 4 (white bars) are control data sets treated in parallel with DTT 1 (light gray bar), DTT 2 through MPD (white bars) and DTT 3 (white bar), respectively. Methods and donor cells are shown at the bottom of the figure. Data are presented as the mean ± SD (Control 1, n = 16; Control 2 and DTT 1, n = 4; Control 3 through DTT 3, n = 3). Control 2 vs. DTT 1 and Control 3 vs. DTT 2, AET, and MPD showed a statistically significant difference at *p* < 0.05 (two-tailed *t*-test). The combination of host strain, vector, and helper is shown at the bottom. Asterisks indicate statistically significant differences: *p < 0.05 and **p < 0.01 (two-tailed *t*-test).

In our previous studies, the basal level of TKC efficiency was adjusted via the number of donor cells to screen recipient mutants with high and low TKC efficiencies [1, 6]. In the course of these studies, we checked that the transformation efficiency increased with an increasing number of donor cells, at least up to the donor:recipient ratio of 50:1. In the simple TKC method, the ratio was approximately 10:1 to 15:1. Thus, we increased the number of donor cells ten times by concentrating the donor cell cultures. The transformation efficiency increased about 30-fold in the low TKC efficiency donor strains (DH10B and S17-1 λ*pir*), and the efficiency was higher than that of HB101 donor transformation using the standard TKC method (Fig 3A). This result shows that these strains are sufficient for practical use if cultures are concentrated before the TKC reaction.

According to previous studies, pretreatment of recipient cells with thiols can increase the transformation efficiency in both chemical and electroporation methods [11, 12]. Although the precise mechanism of the influence of thiols on yeast cells is not clear, an increase in cell permeability in the presence of thiols has been reported [13]. If T4SS of RK2 plasmid does not penetrate the plasma membrane of recipient yeast cells, thiol treatment is expected to positively affect TKC efficiency. Examining this possibility, we observed that using a 30-min treatment with dithiothreitol (DTT) added directly into the recipient cultures increased the transformation efficiency 3-fold over that of non-treated controls (Fig 3B, lanes Control 2 vs. DTT 1). However, compared to another non-treated control data set including results from other independent experiments, the transformation efficiency of this method was two-fold higher without statistical significance (lanes Control 1 vs. DTT 1). To further examine the effect of thiols, we used three different thiols (DTT, 2-Aminoethanethiol [AET] and 3-Mercapto-1,2-propanediol [MPD]) using the adjusted TKC method. As shown on Fig 3B, all thiols examined increased the transformation efficiency by one order of magnitude, a statistically significant difference over that of non-treated controls (lanes Control 1, 2, and 3 vs. DTT 2 to MPD). There was no statistically significant difference in this effect between thiols. DTT treatment of the recipient cells provided sufficient transformation efficiency using S17-1 λ*pir* to warrant the use of this strain (lanes Control 4 vs. DTT 3).

## Discussion

Our results demonstrate that TKC shuttle vectors can be easily introduced into yeast by simply mixing *E*. *coli* and *S*. *cerevisiae* cultures at stationary phase, providing a simple, timesaving method. From another perspective, this result also indicates that trans-kingdom horizontal DNA transfer may have occurred more frequently in the natural environment than predicted, as we suggested previously [1].

In eukaryotic genetics, changing a recipient phenotype by gene introduction is generally called as “transformation,” and “conjugation” means the act of pairing a male and female for reproductive purposes. For these reasons, the term “transformation” is used in this report. Based on bacterial genetics, however, it might be better to use “conjugation” because TKC is driven by a bacterial conjugal transfer system, and because the gene transfer machinery is more strictly classified into three groups, transformation, conjugation, and transduction.

Transformation of *S*. *cerevisiae* using lithium acetate (LiAc) has been the most commonly used method since it was first reported by Ito *et al*. [14], and various modified methods have been established, including a simple method [15]. The LiAc method can transform intact cells without preparing protoplasts. However, it still requires several steps and reagents for both preparation of vector DNA and the transformation itself. The TKC-mediated transformation method reported here is simpler, requiring only the mixing of a small volume of donor and recipient cultures directly. This method would be particularly advantageous for screening a large number of samples, as with interactome analysis using a yeast two-hybrid (Y2H) system. High receptivity strains may be preferred for this purpose, although the TKC method is applicable to a variety of yeast strains (Fig 2B; S1 Table). The introduction of an *SSD1* gene knock out mutation in the EGY48 strain, which is often used for Y2H, successfully converted this strain into a high receptivity strain [1].

The simplified TKC method could also be used for yeast strains that are sensitive to heat shock and/or chemical reagents such as polyethylene glycol. This TKC method is a recipient-friendly method because it does not lead to cell death [1]. Mutants for decreased transformation efficiency induced by TKC are not the same as those induced by chemical transformation methods [6, 16]. These methods may thus compensate for each other’s defects. The TKC method requires dedicated vectors that include an *oriT* (or *oriT/mob*) region and donor bacterial strains with T4SSs to mobilize the vectors. Most importantly, this method is never applicable when a DNA fragment is intended to be introduced directly into recipient cells.

Improving transformation efficiency is desirable for any transformation method. To make the best use of TKC-mediated yeast transformation, methods for increasing transformation efficiency should not increase the complexity of the procedure. We successfully achieved an increase in the transformation efficiency either by increasing the amount of donor cells or by treating recipient cells with thiols (Fig 3). The former will be more preferentially applied in practical use because it adds only one step on the simplest method and is easily applicable to large sample number experiments. The use of thiols also keeps the procedure simpler than that of other transformation methods. In our previous study, we observed that the addition of mitochondrial function inhibitors to a solid medium increased the competence of recipient yeast [1]. Accordingly, we added erythromycin along with DTT to liquid yeast medium to determine the effects on transformation efficiency. No clear synergistic effect was observed in our preliminary data (data not shown). Clues as to the improvement of transformation efficiency may be obtained from functional analysis of low and high TKC receptivity mutants isolated previously [1, 6].

Another remaining plan to improve this method is to enrich the vector content and make it available non-commercially. Our TKC vectors, with several prototrophic markers, for Y2H have been deposited in a public bioresource bank (NBRP-Yeast, http://yeast.lab.nig.ac.jp/nig/index_en.html, ID: BYP7570-7577). We hope that these vectors will be used, further improved, and deposited by participants.

## Conclusion

The trans-kingdom conjugation-mediated transformation method described here may be the simplest, most practical yeast transformation method. This method is also low-cost and timesaving. To be accepted as a general method, enrichment of TKC vectors and donor *E*. *coli* strains is critically important.

## Supporting Information

S1 Fig Distribution of transformation efficiency in chemical and TKC transformation experiments. (TIFF)

S1 Table Summary of results of a simple TKC-mediated transformation method. (DOCX)
