# Supplementary figures and images for: A Fast and Practical Yeast Transformation Method Mediated by Escherichia coli Based on a Trans-Kingdom Conjugal Transfer System: Just Mix Two Cultures and Wait One Hour
Source: PLoS One. 2016 Feb 5;11(2):e0148989. doi: 10.1371/journal.pone.0148989 (PMC4744038; doi:10.1371/journal.pone.0148989)

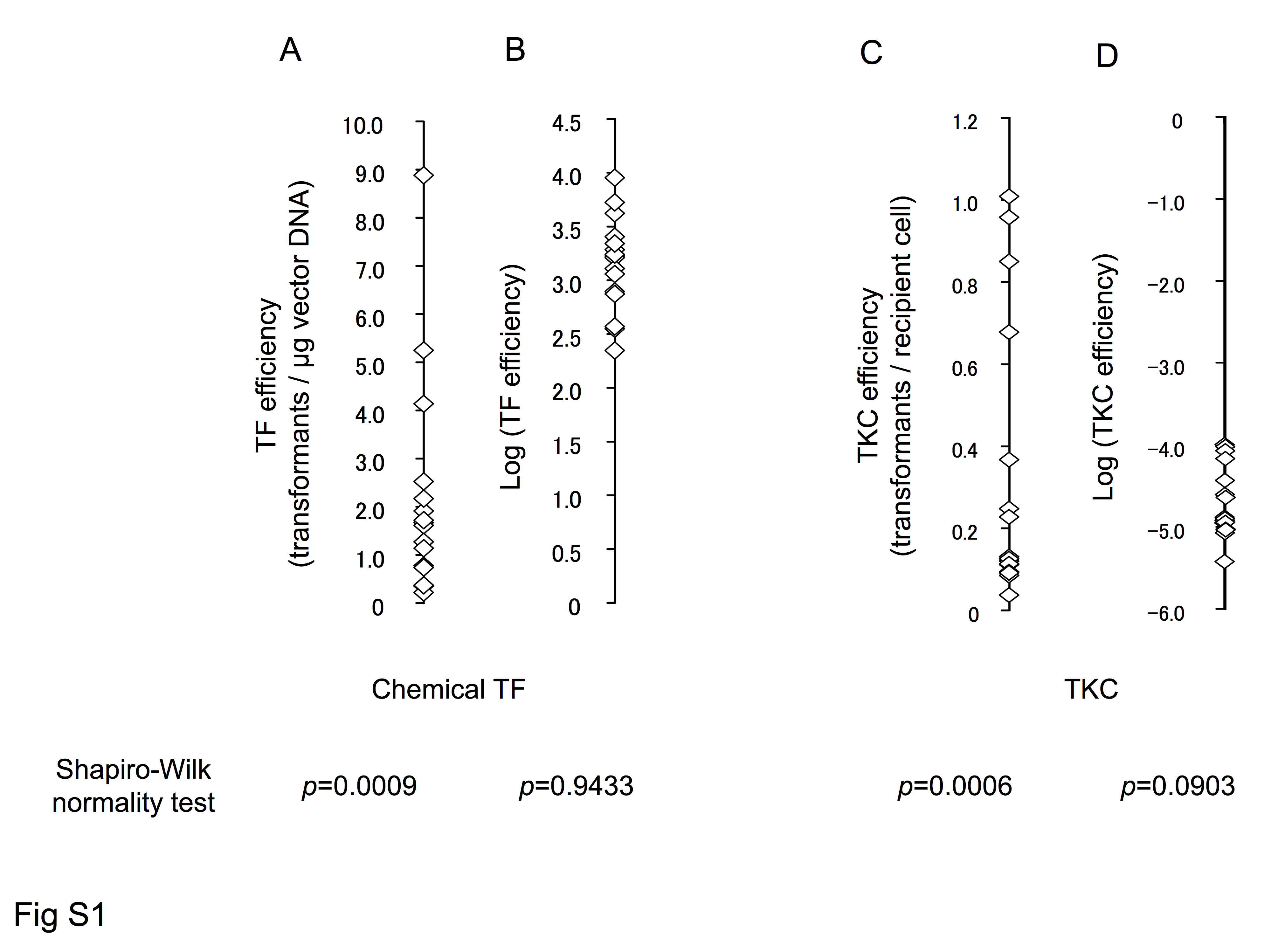

Supplement: S1 Fig — (TIFF) [file pone.0148989.s001.tiff]
