# Supplementary material for: A Fast and Practical Yeast Transformation Method Mediated by Escherichia coli Based on a Trans-Kingdom Conjugal Transfer System: Just Mix Two Cultures and Wait One Hour
Source: PLoS One. 2016 Feb 5;11(2):e0148989. doi: 10.1371/journal.pone.0148989 (PMC4744038; doi:10.1371/journal.pone.0148989)
Supplement: S1 Table — (DOCX) [file pone.0148989.s002.docx]

| **Table S1. Summary of results of a simple TKC-mediated transformation method** | | | | | | | | | | |
| --- | --- | --- | --- | --- | --- | --- | --- | --- | --- | --- |
| **Donor (*E. coli*)** | | | | **Recipient**  **(*S. cerevisiae*)** | **Trials**  **(n)** | **Transformants**  **/100 µl reaction** | | **Average of Log_10_ converted values** | |  |
| **Host strain** | **TKC vector** | **Helper** | **Culture**  **status** |  |  | **Max.** | **Min.** | **Transformants**  **/100 µl reaction** | **Transformants**  **/recipient cell** |  |
| HB101 | pAY205 | pRH210 | Liquid | BY4742 | 16 | 272 | 4 | 1.72 ± 0.50 | −4.69 ± 0.44 |  |
| HB101 | pAY205 | pDPT51 | Liquid | BY4742 | 6 | 716 | 8 | 1.77 ± 0.71 | −4.58 ± 0.72 |  |
| HB101 | pRS316::*oriT*^P^ | pRH220 | Liquid | BY4742 | 10 | 312 | 32 | 2.13 ± 0.30 | −4.22 ± 0.32 |  |
| DH10B | pRS316::*oriT*^P^ | pRH220 | Liquid | BY4742 | 3 | 38 | 6 | 1.12 ± 0.41 | −5.53 ± 0.64 |  |
| DH10B | pRS316::*oriT*^P^ | pRH220 | 10x Liquid | BY4742 | 3 | 638 | 314 | 2.64 ± 0.16 | −3.93 ± 0.15 |  |
| S17-1 λ*pir* | pAY205 | - | Liquid | BY4742 | 3 | 48 | 0 | *NC | *NC |  |
| S17-1 λ*pir* | pRS316::*oriT*^P^ | - | Liquid | BY4742 | 4 | 22 | 2 | 0.89 ± 0.52 | −5.47 ± 0.64 |  |
| S17-1 λ*pir* | pRS316::*oriT*^P^ | - | 10x Liquid | BY4742 | 3 | 1388 | 598 | 2.95 ± 0.18 | −3.60 ± 0.19 |  |
| HB101 | pAY205 | pRH210 | Liquid | YNN281α | 3 | 856 | 512 | 2.85 ± 0.12 | −3.42 ± 0.09 |  |
| HB101 | pAY205 | pDPT51 | Liquid | YNN281α | 6 | 3692 | 576 | 2.98 ± 0.39 | −3.15 ± 0.20 |  |
| HB101 | pRS316::*oriT*^P^ | pRH220 | Liquid | YNN281α | 4 | 3160 | 668 | 3.18 ± 0.31 | −2.93 ± 0.15 |  |
| HB101 | pAY205 | pRH210 | Liquid | NCYC3623 | 3 | 92 | 8 | 1.58 ± 0.59 | −4.05 ± 0.79 |  |
| HB101 | pAY205 | pDPT51 | Liquid | NCYC3625 | 3 | 352 | 20 | 2.08 ± 0.68 | −4.04 ± 0.59 |  |
| HB101 | pAY205 | pDPT51 | Liquid | NCYC3627 | 3 | 12 | 0 | *NC | *NC |  |
| HB101 | pAY205 | pDPT51 | Liquid | NCYC3630 | 3 | 300 | 16 | 2.00 ± 070 | −4.14 ± 0.70 |  |
| HB101 | pAY205 | pDPT51 | Liquid | NCYC3631 | 3 | 36 | 0 | *NC | *NC |  |
| HB101 | pAY205 | pRH210 | Solid | BY4742 | 6 | 76 | 2 | 1.17 ± 0.65 | −5.32 ± 0.70 |  |
| HB101 | pRS316::*oriT*^P^ | pRH220 | Solid | BY4742 | 4 | 88 | 18 | 1.56 ± 0.29 | −4.76 ± 0.47 |  |
| HB101 | pAY205 | pRH210 | Liquid | BY4742  (**Control 2) | 4 | 92 | 20 | 1.55 ± 0.32 | −4.89 ± 0.17 |  |
| HB101 | pAY205 | pRH210 | Liquid | BY4742  (**DTT 1) | 4 | 88 | 72 | 1.98 ± 0.05 | −4.43 ± 0.20 |  |
| *Not calculated; inclusion of data that were less than the detectable threshold. | | | | | | | | | |  |
| **Please refer to Figure 3. | | | | | | | | | |  |
